# Puzzling parrotfishes: Radiocarbon age validation and updated longevity estimates for western Atlantic species in support of sustainable fisheries management

**DOI:** 10.1371/journal.pone.0302854

**Published:** 2024-05-09

**Authors:** Jesus M. Rivera Hernandez, Virginia R. Shervette

**Affiliations:** 1 Department of Biology and Geology, Fish/Fisheries Conservation Lab, University of South Carolina Aiken, Aiken, SC, United States of America; 2 University of South Carolina, Marine Sciences, Columbia, SC, United States of America; MARE – Marine and Environmental Sciences Centre, PORTUGAL

## Abstract

For management efforts to succeed in Caribbean fisheries, local fishers must support and be willing to comply with fishing regulations. This is more likely when fishers are included in a stock assessment process that utilizes robust scientific evidence, collected in collaboration with fishers, to evaluate the health of fish stocks. Caribbean parrotfishes are important contributors to coral reef ecosystem health while also contributing to local fisheries. Scientifically robust stock assessments require regional species-specific information on age-based key life history parameters, derived from fish age estimates. Evaluation of the accuracy of age estimation methods for fish species is a critical initial step in managing species for long-term sustainable harvest. The current study resulted from a collaborative research program between fish biologists and local fishers investigating age, growth, and reproductive biology of the seven parrotfish species landed in U.S. Caribbean fisheries; specifically, we validated age estimation for stoplight parrotfish *Sparisoma viride* and queen parrotfish *Scarus vetula*. This is the first study to directly validate age estimation for any parrotfish species through analysis of Δ^14^C from eye lens cores. Our age estimation validation results show that enumeration of opaque zones from thin sections of sagittal otoliths for a *Sparisoma* and a *Scarus* species provides accurate age estimates. The oldest stoplight parrotfish and queen parrotfish in the Δ^14^C age estimation validation series were 14 y and 16 y; while the oldest stoplight parrotfish and queen parrotfish we aged to-date using the Δ^14^C validated age estimation method were 20 y and 21 y, respectively. Fish longevity (maximum age attained/life span) is a key life history parameter used for estimation of natural mortality, survivorship, and lifetime reproductive output. Past reviews on parrotfishes from the Pacific and Atlantic concluded that most Caribbean/western Atlantic parrotfish species are relatively short-lived with estimated maximum ages ranging from 3–9 y. However, information from our collaborative research in the U.S. Caribbean combined with recently published age estimates for Brazilian parrotfish species indicate that many western Atlantic parrotfishes are relatively long-lived with several species attaining maximum ages in excess of 20 y.

## Introduction

Parrotfishes are integral contributors to the ecosystem function and maintenance of shallow water coral reefs due to their roles as algal consumers and recyclers of coral skeletal material into copious amounts of sediment [1–4]. Parrotfishes also are important food fishes targeted by artisanal (small-scale) commercial fisheries throughout much of the Caribbean [5–8]. Despite their importance in Caribbean reef fisheries, parrotfishes are considered data-deficient/data-poor in terms of fisheries management due to a lack of species-specific information regarding population demographics. In the U.S. Caribbean, there is a critical need for documenting basic life history parameters of Caribbean parrotfishes so that fisheries scientists, in collaboration with local fishers and local resource managers, can conduct scientifically rigorous stock assessments and then implement relevant management strategies to ensure the long-term sustainable harvest of parrotfishes. The determination of population age structure is an essential component in addressing life history information gaps for Caribbean parrotfishes. Age estimates of individuals in a population are used with corresponding fish length data and information on sex to calculate growth rates, mortality, age/size at sexual maturity, and for sequential hermaphrodites such as parrotfish species, age/size at sexual transition; all are integral life history parameters used in fisheries management [6,9–12]. Longevity (maximum age/life span) is another important age-based fisheries management parameter that is used to obtain estimates related to survivorship and mortality [10–12].

Many coral reef fish species have complex sexual ontogenies which can influence size and sex specific growth patterns [13–17]. Parrotfishes in particular exhibit a combination of complex life history patterns and reproductive strategies [13,18–21]. Additionally, several studies have noted a decoupling of size and age in parrotfish species emphasizing that size can be a poor estimate of age in terms of understanding demographic patterns related to life history [15,19,22,23]. Ultimately, age data, preferably derived from a validated age estimation method, are essential to document the biology and ecology of parrotfish populations.

The most common means of ageing marine bony fishes is by enumeration of growth increments in otoliths. However, otolith increments in tropical reef fishes can be relatively difficult to visualize or interpret which may be due to a combination of environmental (e.g., lower annual temperature variability) and biological (e.g., complex life histories) factors [24–26]. Therefore, age estimation validation can be critically important to establish the accuracy of an ageing method for tropical reef fish species [27,28].

Methods used to enumerate annual growth increments in otoliths have been validated through numerous approaches; however, one of the best-suited methods to validate age estimates of medium to long-lived species is application of a regional bomb radiocarbon (^14^C) time series [28–32]; utilization of ^14^C is a well-established, scientifically rigorous method for age estimation validation [30,32–35]. The bomb-derived ^14^C was introduced into the atmosphere from testing of nuclear weapons starting in 1945 until the early 1960s [36,37]. This caused a rapid increase in marine ^14^C, through air-sea diffusion, that peaked in the mid-1970s to the early 1980s and then a subsequent steady decline of ^14^C in marine surface waters occurred (< 100 m depth) [19,29,32,38,39]. Region-specific bomb radiocarbon time series have been established through analysis of biogenic CaCO_3_ from hermatypic corals and fish otoliths and applied extensively to validate ageing methods for reef fishes that had birth years (hatch years) in the 1950s and 1960s during the period of rapid rise in oceanic ^14^C [30,33,40–44] and more recently, the post-peak ^14^C decline period has been applied to validate bony fish age estimates of younger and more recently collected fishes [19,25,26,29,32,45–48]. Time-specific ^14^C records from shallow marine waters provide regional reference chronologies that are used to evaluate fish age estimates through comparison of fish ^14^C measured in otolith cores and eye lens cores that formed during the first few months of life [26,29,34,44,46]. Shervette et al. [32] published a reference ^14^C time series for the north Caribbean and it has been used to successfully validate age estimation methods for several reef-associated fishes from the region, including mutton snapper *Lutjanus analis* [32], yellowtail snapper *Ocyurus chrysurus* [48,49]. Queen snapper *Etelis ocula*tus [25], red hind *Epinephelis guttatus* [32], white grunt *Haemulon plumieri* [32], queen triggerfish *Balistes vetula* [26], and hogfish *Lachnolaimus maximus* [47].

Fish otoliths are composed principally of aragonite and other forms of biogenic CaCO_3_, which are metabolically inert once formed, with ^14^C incorporated into otoliths from dissolved inorganic carbon from the surrounding seawater and dietary sources [34,50]. Precise extraction of otolith core material for species with characteristically small, thin, fragile otoliths can be difficult and often requires access to computerized micromilling systems [25,45,51]. However, several studies recently demonstrated that eye lens cores contain archived chemical isotopic signatures from early life [26,52–55] and have been successfully used to determine the radiocarbon signature a fish experienced during early life which enabled age estimation for a deepwater shark species [56] and age validation for populations of several tropical marine fish species [25,26,47–49]. Eye lens cores can be used as a source of birth year (hatch year) carbon signatures in bomb radiocarbon age validation efforts because eye lenses begin formation prior to hatching [57,58], grow throughout the life of a fish [52,57,58], and consist of carbon-rich optical proteins that become metabolically inert shortly after formation and are deposited in successive, concentric layers [54,55,57,58].

**Fig 1:**
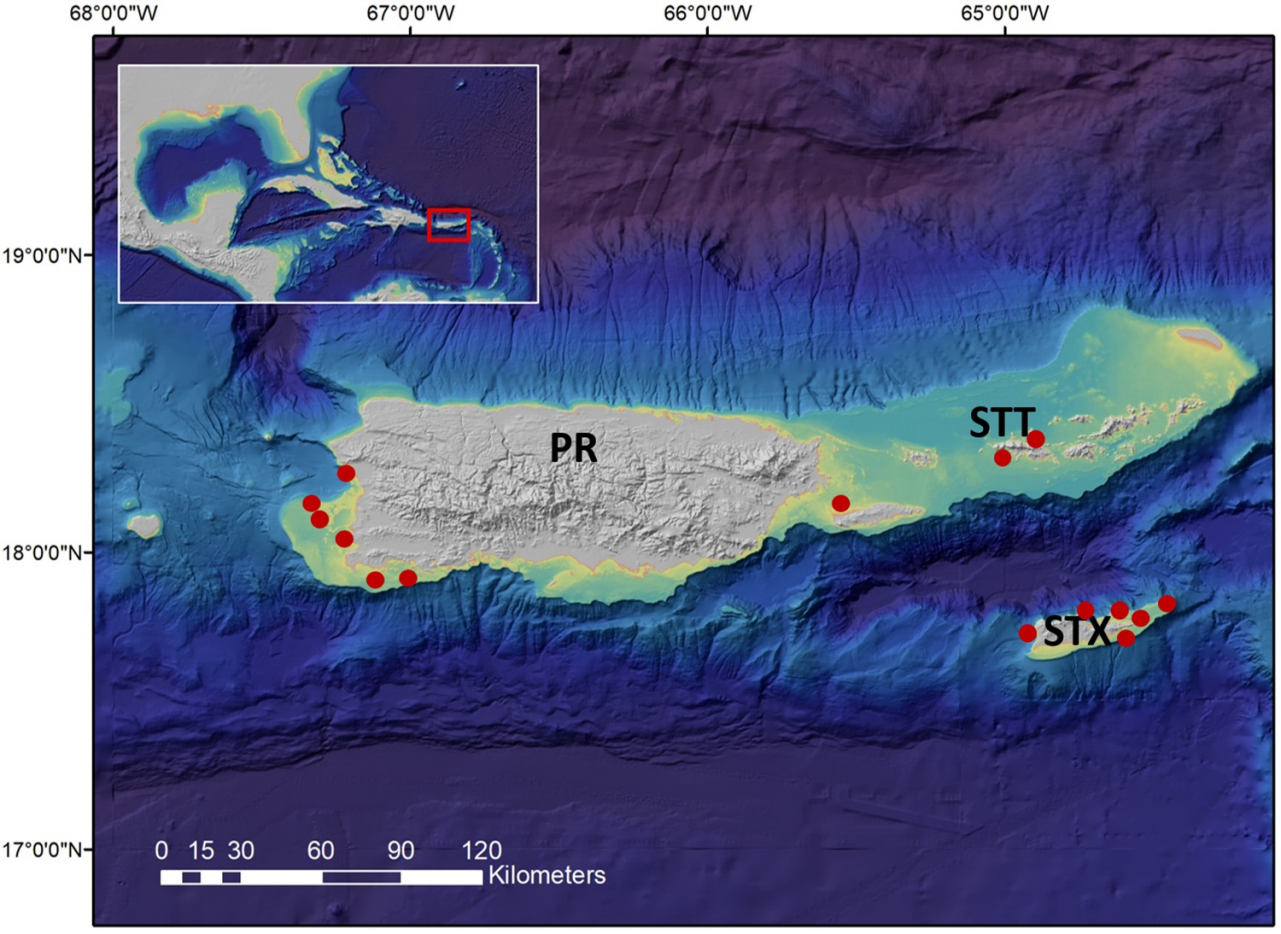
Sampling region for Caribbean parrotfishes. Map represents the U.S. Caribbean study region and includes the major islands of Puerto Rico (PR), St. Thomas (STT), and St. Croix (STX). Red circles indicate sites of collections. The map layer used to generate this figure is from NOAA National Centers for Environmental Information and provided without restriction by the U.S. Government.

In the U.S. Caribbean (Fig 1), seven parrotfish species are landed in the reef fish fisheries of Puerto Rico (PR) and the U.S. Virgin Islands (USVI): stoplight parrotfish *Sparisoma viride*, redtail parrotfish *Sp*. *chrysopterum*, redband parrotfish *Sp*. *aurofrenatum*, yellowtail parrotfish *Sp*. *rubripinne*, princess parrotfish *Scarus taeniopterus*, striped parrotfish *Sc*. *iseri*, and queen parrotfish *Sc*. *vetula* [5,7,59–62]. In general, Caribbean parrotfishes are described as sequential hermaphrodites displaying protogyny; an individual is first a female and then transforms into a male but does not function simultaneously as both [13,20,21]. In some species, primary males (male not derived from females) have been observed (males that transitioned from females are called secondary males) [13]. Additionally, presumed gonochoristic-like females (females that do not appear to transform to males) may occur in some populations [63], but this must be confirmed with age data, since the presence of large females does not necessarily equate to old females. Most parrotfish species also exhibit a complex sequence of ontogenetic changes in color patterns associated with sexual identities; all but one of the West Atlantic *Scarus* and *Sparisoma* species (midnight parrotfish *Sc coelestinus*) exhibit two distinct color patterns as adults: initial color phase and terminal color phase [63].

Parrotfish species in the U.S. Caribbean need to undergo stock assessments; past attempts to assess parrotfish populations were incomplete due to a lack of basic life history information [62]. Thus, the overall goal of this study was to utilize ^14^C to validate the age estimation method of enumerating growth zones from sagittal otoliths of parrotfishes from the north Caribbean as a first step towards documenting population demographics and investigating species-specific life history strategies. Validation of the age estimation method for parrotfishes ensures that accurate ages can be used to compute population parameter estimates. Specific objectives were primarily to utilize ^14^C (reported as Δ^14^C) from eye lens cores to assess age estimation accuracy for stoplight parrotfish and queen parrotfish. A secondary objective of this paper was to provide an updated summary of longevity (maximum age/life span) estimates for parrotfish species from the western Atlantic. This is because several past studies that investigated demographic patterns of parrotfishes from the Pacific and Atlantic emphasized that many species are relatively short-lived [15,22,64–66]. Molina-Ureña [63] conducted an extensive literature review on biology and ecology of western Atlantic parrotfishes and noted that lifespan of Atlantic species ranged from 3–9 y, although since then, additional studies from the Caribbean/western Atlantic have documented maximum age estimates of 16 y and older for many species. These more recent observations emphasize a need to re-evaluate our understanding concerning overall patterns of longevity for parrotfishes from the western Atlantic.

## Materials and methods

Fish samples obtained by the authors of this study and reported on here were collected and handled in strict accordance within the guidelines of the U.S. Government Principles for the Utilization and Care of Vertebrate Animals Used in Testing, Research and Training.

(https://olaw.nih.gov/sites/default/files/PHSPolicyLabAnimals.pdf). This research was conducted under USCA IACUC protocol #053012-BIO-04.

### Age estimation for Caribbean parrotfishes

Fish samples with otoliths and eyes selected for this validation study were obtained from an archived collection of stoplight parrotfish and queen parrotfish specimens sampled from Puerto Rico (PR), St. Thomas/St. John (STT/J), and St. Croix (STX; Fig 1) by the Fish/Fisheries Conservation Lab at University of South Carolina Aiken (USCA FishCon Lab) as part of a large, ongoing research program designed to fill in critical life history information data-gaps for U.S. Caribbean parrotfishes in collaboration with local fishers. Relevant information collected for each fish sample included length measurements recorded in the field (mm), color phase, date of collection, and the general location of capture. Whole eyes were dissected from fish collected during sampling efforts, wrapped in foil, and stored at -20° C.

**Fig 2:**
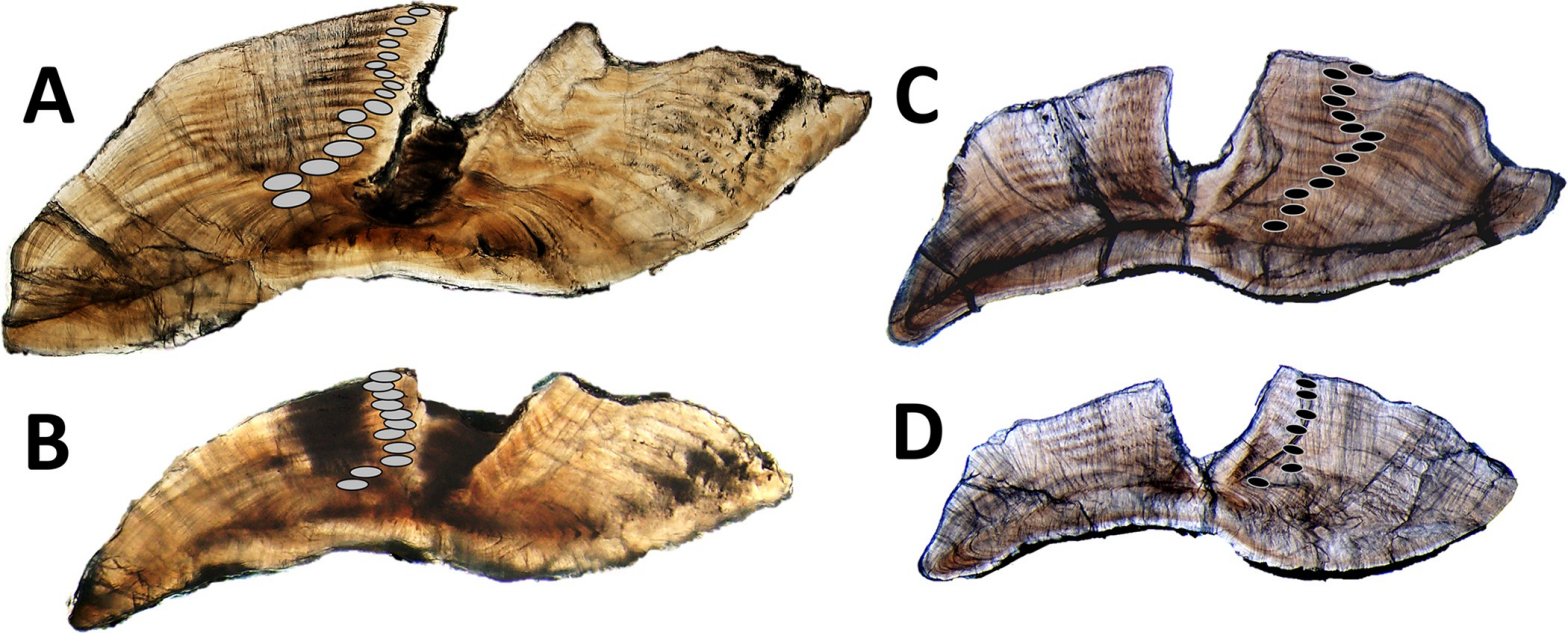
Annotated otolith sections for Caribbean *Scarus* and *Sparisoma* parrotfish species from the western Atlantic. A. Queen parrotfish from U.S. Caribbean included in the lens core validation samples (terminal color phase male, 369 mm FL, 16 y). B. Princess parrotfish from U.S. Caribbean (terminal color phase male, 314 mm TL, 11 y); age of sample originally reported in Jones et al. [67]. C. Stoplight parrotfish from U.S. Caribbean included in the lens core validation samples (initial color phase female, 304 mm FL, 14 y). D. Stoplight parrotfish from U.S. Caribbean included in the lens core validation samples (terminal color phase male, 369 mm FL, 7 y).

Sagittal otoliths were processed for age estimation following the methods described previously in Jones et al. [20]. One sagittal otolith from each fish sample was embedded in epoxy, then otoliths were sectioned transversely through the nucleus to a thickness of ~ 0.3 mm using a low-speed saw with a diamond-edged blade. Otolith sections were mounted on glass slides and covered with a clear mounting media. Otolith section slides were read using transmitted light at a magnification of 20-40x. Two independent readers (each with over 10 y of experience in tropical fish age estimation work) recorded the number of opaque zones visible in otoliths sections for each fish sample (one pair of alternating translucent and opaque zones equals one increment; Fig 2). If disagreement between readers occurred, then the otolith sections were re-evaluated by both readers at the same time and a consensus opaque zone count was obtained as the final age estimate. Increment counts were assessed without knowledge of fish size or time of year that the sample was collected.

Specimens used for stoplight parrotfish and queen parrotfish age validation consisted of randomized selection of individuals from the USCA FishCon lab’s archived parrotfish samples with both otoliths and eyes available for the following groupings: youngest fish for each species; initial color phase male samples; oldest female samples, oldest male samples, individuals binned in age class groups across the full range of ages available when the current study on age validation was conducted.

### Bomb radiocarbon age estimation validation

We determined the target diameter of the eye lens core region (hereafter referred to as “lens core”) for obtaining the Δ^14^C signature that an individual fish experienced during the first ~ 6 months of life was approximately 1 mm for the two parrotfish species. We based this on examination of the eye lens diameter of age-0 fish opportunistically collected with a castnet in July 2019. Analysis of age-0 stoplight parrotfish (n = 4; mean SL = 73 mm, 6.9 SD) eye lenses indicated a mean dry eye lens diameter of 0.9 mm (0.11 SD) and mean mass of 0.8 mg (0.12 SD). Analysis of age-0 queen parrotfish (n = 2; mean SL = 91 mm, 1.4 SD) indicated a mean dry eye lens diameter of 1.0 mm (0.10 SD) and mean mass of 0.9 mg (0.11 SD). Subsequent examination of presumed daily increments in otoliths for two of the stoplight parrotfish juveniles and for both queen parrotfish juveniles (which was part of a separate examination on juvenile parrotfish habitat use) confirmed that fish were approximately six months old; stoplight parrotfish juvenile otoliths contained 150–183 presumed daily increments and queen parrotfish juvenile otoliths contained 158–191 presumed daily increments.

**Fig 3:**
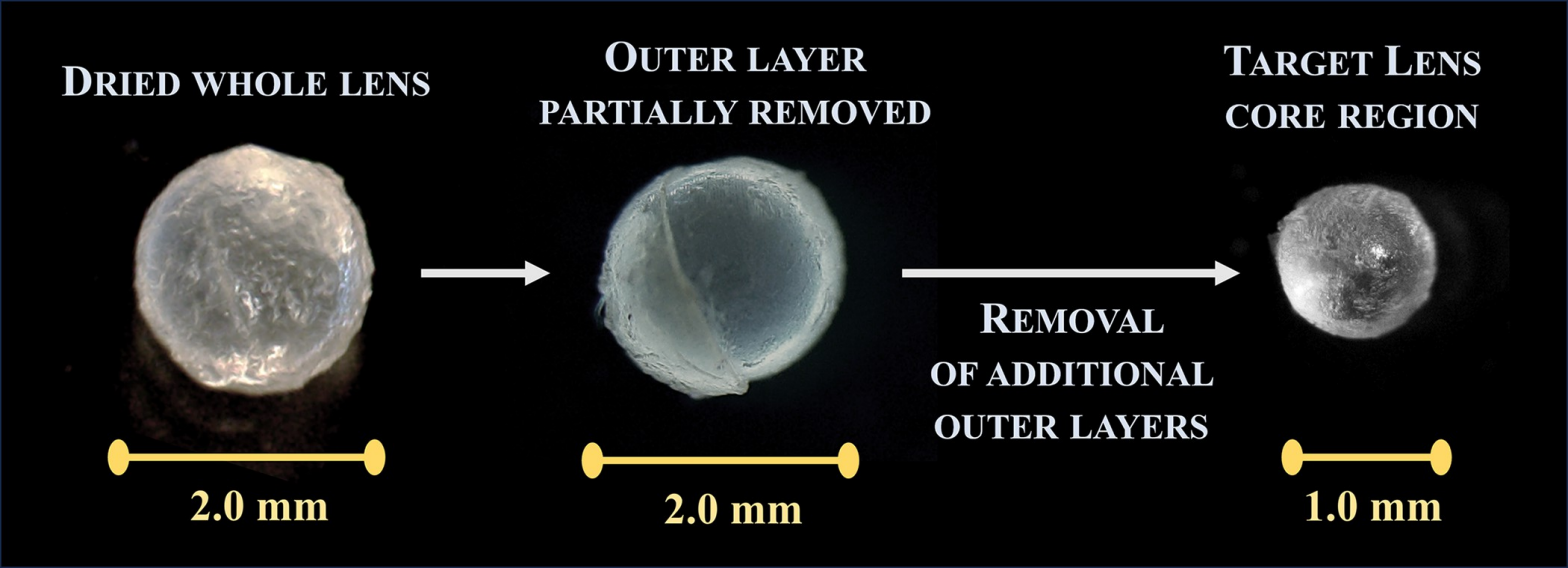
Visual summary of the eye lens pealing process. Starting with an air-dried whole parrotfish eye lens, the concentric layers are carefully removed until reaching the lens core.

Forceps and glassware used to obtain and store eye lens cores for Δ^14^C analysis were pretreated to prevent carbon contamination as described in Shervette and Rivera Hernández [26]. For each of the age estimation validation fish samples, whole eyes were thawed at room temperature then eye lenses extracted. Next, lenses were dried in pretreated glass petri dishes and then peeled by removing the concentric layers until the target core diameter (1 mm) was reached (Fig 3). Each lens core was weighed to the nearest 0.1 mg. In some fish, a single core did not provide sufficient mass for robust isotopic analysis (minimum mass for high precision results = ~0.6 mg), in which case we obtained the equivalent-sized core from the second eye lens of the same fish and combined the two lens cores [54]. Lens cores were placed in pre-treated glass vials for shipment and cores were analyzed for Δ^14^C via accelerator mass spectrometry at the National Ocean Sciences Accelerator Mass Spectrometry facility at Woods Hole Oceanographic Institute (www.whoi.edu/nosams/radiocarbon-data-calculations).

“Birth year” (hatch year/year of formation of eye lens core) for each sample was computed by subtracting the otolith-based estimated age of a sample (the number of opaque zones counted within the otolith sections) from the year of sample collection. For example, a fish caught in 2019 with an estimated age of 14 y (based on the otolith section increment count) would have a birth year of 2005. Stoplight parrotfish spawn year round [17], with peak spawning documented from February-April for the north Caribbean [21]. Peak spawning month for queen parrotfish, based on histological analysis of gonads from mature females collected from 2015–2022 (as part of our ongoing parrotfish life history work) was also February-April. We adjusted the birth year value for each sample by +0.5 to incorporate the timing of peak spawning and the midpoint in time that the lens core formed [26]. In the example above of the fish with an estimated birth year of 2005: final adjusted birth year of 2005.5 = 2005 + 0.25 (peak spawning month of March = 3/12) + 0.25 (midpoint in time that the lens core region formed [6 months/2]).

**Fig 4:**
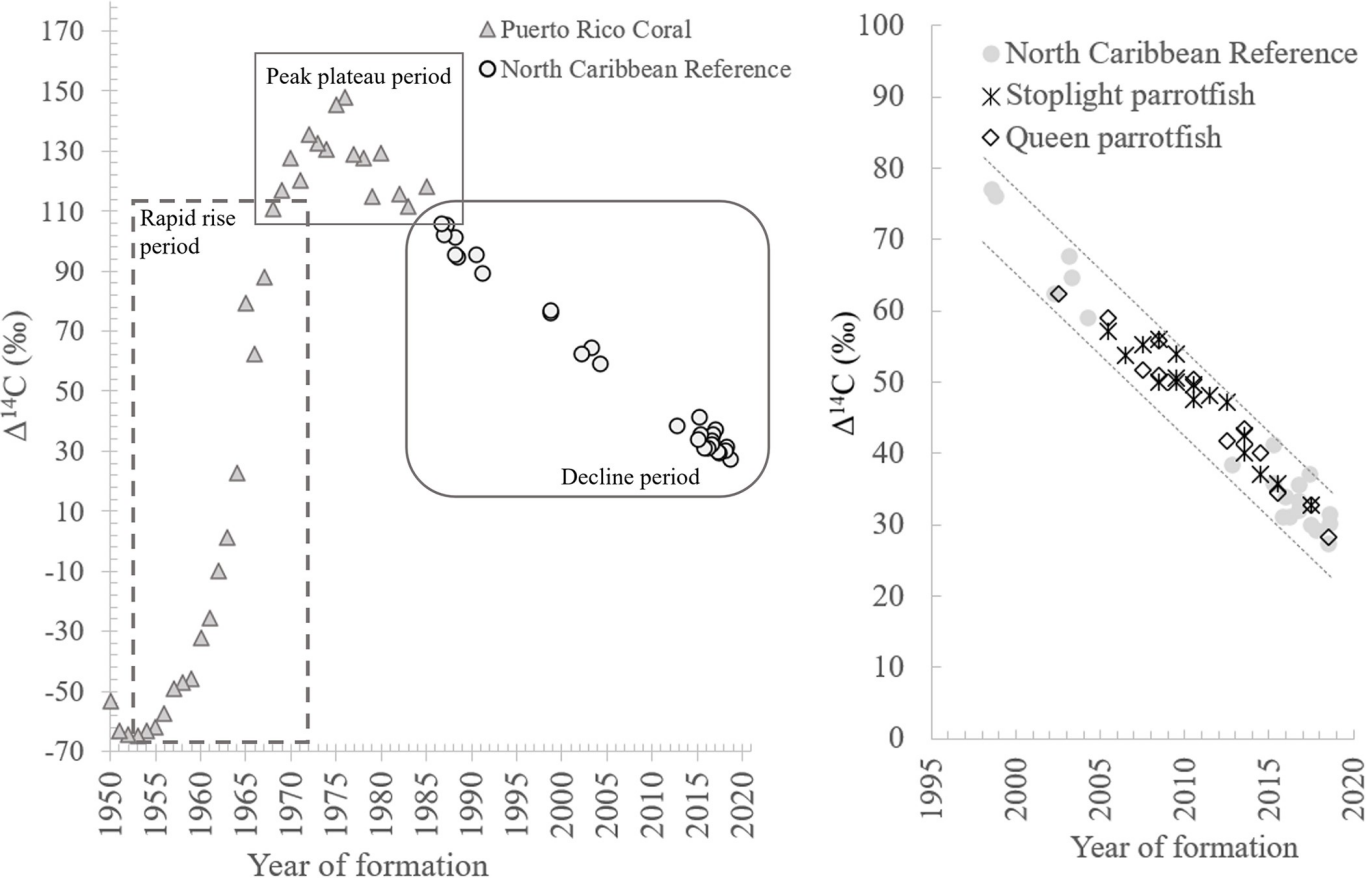
Regional Caribbean reference Δ^14^C time series and results for parrotfishes. Radiocarbon time series for waters of the north Caribbean (left) including data obtained from coral (Kilbourne et al. 2007) and the North Caribbean Reference Δ^14^C (Shervette et al. 2021). Results from Δ^14^C lens cores for parrotfish samples (right) overlayed on the North Caribbean Reference Δ^14^C time series; dashed lines represent upper and lower 95% prediction intervals for the reference Δ^14^C regression model.

The accuracy of the age estimation method used for the two parrotfish species was evaluated in two ways. First, similar to previous bomb radiocarbon fish age validation studies, including one focused on Pacific parrotfishes [19,29,68], we examined the concordance of the validation series of lens core Δ^14^C –estimated birth year for each species with the trends established by the north Caribbean Δ^14^C reference time series. This was done through comparing the series of lens core Δ^14^C –estimated birth year data for each species with the reference time series using ANCOVA to evaluate if significant differences occurred between regression slopes and intercepts [19]. Second, we used separate sum of squared residuals (SSR) bias analysis [26,32,44] for each species. Estimated birth years and corresponding Δ^14^C of stoplight and queen parrotfish eye lens cores were overlaid on the north Caribbean reference Δ^14^C time series (Fig 4) [32]. Birth years derived from original age estimates represented an age bias of 0 (null model), age bias models of +1, +2, +3 shifted age estimates older, and age bias models of -1, -2, -3 shifted age estimates younger. For each model, using the north Caribbean Δ^14^C reference time series linear regression equation (y = 4680–2.30x) [32], we computed the sum of squared residuals (SSR) from observed eye lens core Δ ^14^C minus predicted values. The model with the lowest SSR is considered the most parsimonious prediction of birth years; if the null model for a species produces the lowest SSR, then the age estimation method is considered accurate [44].

## Results

For stoplight parrotfish, lens core Δ^14^C was obtained for 17 samples with estimated ages ranging from 1–14 y and corresponding birth years ranging from 2005–2017 (Table 1). The oldest stoplight parrotfish included in the validation series was an initial color phase female (14 y; 304 mm FL) collected from waters of St. Croix, USVI (Table 1 and Fig 2). Female stoplight parrotfish analyzed for Δ^14^C ranged in size from 127–334 mm FL and age from 1–14 y. Terminal color phase males ranged in size from 292–371 mm FL and age from 5–12 y. The youngest male in the validation series was an initial color phase individual with a length of 307 mm FL and age of 4 y (Table 1).

**Table 1:** Stoplight parrotfish and queen parrotfish eye lens core validation samples.

Species	FishID	Size (mm)	Color phase Sex	Year Caught	Age	Birth year	δ^13^C	Δ^14^C	σ
Stoplight	STX-SPF01	257 SL/304 FL	Initial Female	2019	14	2005.5	-15.88	57.21	2.00
Stoplight	STX-SPF02	321 SL/371 FL	Terminal Male	2018	12	2006.5	-15.54	53.74	2.30
Stoplight	STX-SPF03	285 SL/334 FL	Initial Female	2018	11	2007.5	-15.93	55.25	2.10
Stoplight	PR-SPF01	297 SL/355 FL	Terminal Male	2019	11	2008.5	-13.85	55.93	2.20
Stoplight	STX-SPF04	285 SL/327 FL	Terminal Male	2018	10	2008.5	-16.31	50.07	2.00
Stoplight	STT-SPF01	316 SL/365 FL	Terminal Male	2019	10	2009.5	-16.48	50.50	2.60
Stoplight	PR-SPF02	273 SL/330 FL	Terminal Male	2019	10	2009.5	-13.49	49.96	2.30
Stoplight	STX-SPF05	265 SL/310 FL	Initial Female	2018	9	2009.5	-16.85	54.02	2.10
Stoplight	PR-SPF03	247 SL/292 FL	Initial Female	2018	8	2010.5	-12.06	49.63	2.30
Stoplight	STT-SPF02	306 SL/360 FL	Terminal Male	2018	8	2010.5	-16.65	47.55	2.00
Stoplight	STT-SPF03	302 SL/357 FL	Terminal Male	2018	7	2011.5	-14.89	48.11	2.50
Stoplight	STT-SPF04	322 SL/369 FL	Terminal Male	2019	7	2012.5	-15.26	47.17	2.20
Stoplight	PR-SPF04	275 SL/329 FL	Terminal Male	2019	6	2013.5	-13.99	39.99	2.10
Stoplight	PR-SPF05	262 SL/299 FL	Terminal Male	2018	5	2013.5	-13.20	42.56	2.10
Stoplight	STT-SPF05	273 SL/310 FL	Terminal Male	2019	5	2014.5	-14.27	37.05	2.00
Stoplight	PR-SPF06	263 SL/307 FL	Initial Male	2019	4	2015.5	-13.88	35.75	2.00
Stoplight	STT-SPF06	103 SL/127 FL	Initial Female	2018	1	2017.5	-14.06	32.73	2.10
Queen	STX-QPF01	309 SL/369 FL	Terminal Male	2018	16	2002.5	-13.96	62.38	2.20
Queen	STX-QPF02	262 SL/322 FL	Initial Transition	2019	14	2005.5	-12.01	58.97	2.30
Queen	STX-QPF03	311 SL/384 FL	Terminal Male	2018	11	2007.5	-12.13	51.65	2.20
Queen	STX-QPF04	282 SL/340 FL	Terminal Male	2018	10	2008.5	-11.68	55.89	2.30
Queen	STX-QPF05	321 SL/395 FL	Terminal Male	2018	10	2008.5	-14.62	51.02	2.10
Queen	STX-QPF06	330 SL/382 FL	Terminal Male	2018	8	2010.5	-13.66	50.46	2.20
Queen	STX-QPF07	310 SL/378 FL	Terminal Male	2019	7	2012.5	-11.86	41.77	2.10
Queen	STX-QPF08	224 SL/274 FL	Terminal Male	2019	5	2014.5	-15.50	40.16	2.10
Queen	STX-QPF09	182 SL/225 FL	Initial Female	2018	5	2013.5	-13.80	43.45	2.80
Queen	PR-QPF01	172 SL/211 FL	Initial Female	2019	4	2015.5	-15.04	34.50	2.60
Queen	STX-QPF10	148 SL/181 FL	Initial Female	2019	2	2017.5	-14.40	32.75	2.10
Queen	STX-QPF11	115 SL/140 FL	Initial Transition	2019	1	2018.5	-12.52	28.28	2.20

A total of 12 queen parrotfish samples had Δ^14^C eye lens core results and ranged in age from 1–16 y with corresponding birth years of 2002–2018 (Table 1). The oldest queen parrotfish included in the validation series was a terminal color phase male (16 y; 369 mm FL) collected from St. Croix, USVI (Table 1 and Fig 3). Females analyzed for Δ^14^C ranged in size and age from 181–225 mm FL and 2–5 y (Table 1). Terminal color phase males ranged in size and age from 274–384 mm FL and 5–16 y. Initial color phase queen parrotfish with gonads transitioning from female to male ranged in size and age from 140–322 mm FL and 1–14 y.

Results for Δ^14^C ranged from 32.73–57.21‰ for stoplight parrotfish, and 28.28–62.38‰ for queen parrotfish (Table 1 and Fig 4). Age estimates for all the stoplight parrotfish and queen parrotfish samples included in the validation series had corresponding birth year estimates versus lens core Δ^14^C that fit within the linear regression 95% prediction intervals of the north Caribbean reference Δ^14^C time series for the decline period (Fig 4).

The three sets of regression relationships (for stoplight parrotfish, queen parrotfish, and the reference time series spanning the relevant portion of the bomb ^14^C decline period) did not differ in their slopes (ANCOVA: F_2,40_ = 2.150; P = 0.130). Using a common slope (b = -2.229), intercepts of the three regressions were not significantly different (F_2,45_ = 0.974; P = 0.381).

The SSR ageing bias analysis results for stoplight parrotfish indicated that the original, unadjusted age estimates (null model) provided accurate ages because they had the lowest SSR at 81 (Table 2). The SSR results for the stoplight parrotfish biased age models ranged from 129 to 953 (Table 2). Similarly, the SSR ageing bias analysis results for queen parrotfish indicated that the null model (unadjusted age estimates) utilized accurate ages because it had the lowest SSR at 62 (Table 2). The SSR results for the queen parrotfish biased age models ranged from 115 to 664 (Table 2).

**Table 2:** Results from ageing bias analysis. Birth year estimates were purposefully biased by +/- 1 to 3 years for each species and then the squared residuals from the predicted north Caribbean reference Δ^14^C time series regression were computed and summed for each age model.

Age Model	Bias applied (y)	Stoplight parrotfish SSR	Queen parrotfish SSR
Null	0	81	62
-1	-1	203	115
-2	-2	493	295
-3	-3	953	603
+3	+3	733	664
+2	+2	346	336
+1	+1	129	136

## Discussion

The current study was the first to directly validate age estimation for parrotfish species through analysis of Δ^14^C from eye lens cores and the first study to validate age estimation for Caribbean parrotfishes utilizing Δ^14^C. Our results show that enumeration of opaque zones from thin sections of sagittal otoliths of *Sparisoma* and *Scarus* species provides accurate age estimates. Utilization of Δ^14^C from fish eye lens cores has several advantages compared to otoliths cores, especially for species with small, fragile otoliths that are generally inhospitable to precise otolith core material extraction. Fish eye lenses are relatively easy to obtain and to process for lens cores compared to obtaining otolith core material which often requires the use of a computerized micromill system. Additionally, many fish species have otoliths that are so small they do not contain enough otolith core material representing birth year Δ^14^C signatures required for precise Δ^14^C analysis [25] or are morphologically shaped such that the otolith core cannot be extracted without contamination from subsequent years beyond the birth year [26].

### Radiocarbon validation of age estimation for parrotfish species

In addition to the current study which validated age estimation for parrotfish species from waters of the western Atlantic, two studies from waters of the Pacific utilized Δ^14^C in age validation efforts for three parrotfish species and a wrasse species [19,51]. The first, Andrews et al. [51], had the stated goal of determining the feasibility of using Δ^14^C to date the otoliths from adult humphead wrasse *Chelinus undulatus* (n = 7) and bumphead parrotfish *Bolbometopon muricatum* (n = 5) and to evaluate the accuracy of age estimates obtained via increment counts in thin sections of sagittal otoliths for the two species. That study focused on large adults in hopes of obtaining fish old enough so that the birth year otolith core Δ^14^C signatures aligned with the Δ^14^C rapid rise period (1950s-1960s; general time period illustrated in Fig 4) of the regional reference time series because that period would provide an extremely narrow range of predicted age estimates (±1–2 y) for the samples based on the Δ^14^C results. However, all seven humphead wrasse and two of the five bumphead parrotfish samples analyzed had estimated birth year Δ^14^C results that when overlaid on the regional Δ^14^C reference series occurred during the 15–20 y Δ^14^C plateau period (1970s-early 1980s; general time period illustrated in Fig 4). Andrews et al. [51] noted “this result is not ideal in terms of validation age” essentially acknowledging the results could not be used to conclusively validate the accuracy of the ageing method. But Andrews et al. (2015) concluded that for humphead wrasse “it is likely that growth zone counting is an accurate method” for age estimation of this species since sagittal otolith sections contained “clean and clearly visible series” of growth zones. For bumphead parrotfish, three of the five samples selected for age validation had estimated birth year-Δ^14^C values that did occur during the rise period of the reference time series. However. two of the samples fell outside of the loess curve prediction intervals for the reference Δ^14^C time series possibly indicating that both fish may have been underage by a few years or that the otolith core samples analyzed for Δ^14^C were contaminated with otolith material that formed later in life. Andrews et al. [51] may not have fully accomplished the goal of validating the age estimation method, but the study demonstrated that bumphead parrotfish can attain ages in excess of 30 y and it also emphasized that it could be challenging to obtain parrotfish otolith core material, even with a computerized micromill system, because of otolith fracturing during the milling process.

The second study from the Pacific that applied Δ^14^C to validate age estimation in parrotfish species focused primarily on documenting growth rates and longevities for five species of parrotfishes that contributed to Hawaiian fisheries [19]. Two of the five species were evaluated for age validation via Δ^14^C: redlip parrotfish *Scarus rubroviolaceus* (n = 23 for Δ^14^C) and spectacled parrotfish *Chlorurus perspicillatus* (n = 21 for Δ^14^C). The estimated birth year-Δ^14^C values from micromilled otolith cores for both species occurred during the Δ^14^C decline period (general time period illustrated in Fig 4) of the regional reference time series. DeMartini et al. [19] utilized ANCOVA to evaluate if the overall linear trends of the two species differed significantly from the Δ^14^C reference decline trend and since no significant results were indicated, the study concluded that the ageing method used for all five parrotfish species provided accurate age estimates. Both redlip parrotfish and spectacled parrotfish had maximum ages of 19 y. From the combined results of our study and DeMartini et al. [19], enumeration of growth increments in sagittal otolith sections from Pacific and West Atlantic parrotfish species appear to provide accurate age estimates.

### Observed trends for stoplight parrotfish and queen parrotfish

The oldest stoplight parrotfish from the lens validation samples was 14 y. Using the validated ageing method from this study, we have documented several stoplight parrotfish from the U.S. Caribbean collected prior to 2018 (which was the year that the large, ongoing collaborative study on documenting life history strategies for Caribbean parrotfishes started saving eyes) that were older than 14 y including an initial color phase female (319 mm FL) collected from STX with an age of 20 y [69]. Van Rooij and Videler [70] utilized repeated visual censuses that included marked fish to estimate growth and mortality rates for stoplight parrotfish across multiple monitoring sites in waters of Bonaire. Results from that study indicated that 10% of stoplight parrotfish >250 mm FL attained an estimated age of 17 y and older with an estimated maximum life span of 25 y for the species [70]. A maximum age of 20 y for stoplight parrotfish from the U.S. Caribbean seems to further support the work of Van Rooij and Videler [70] in that the species can attain a maximum age of 20 y or more. Reef parrotfish *Sparisoma amplum*, the Brazilian sister species of stoplight parrotfish [71], appears to attain a maximum age of at least 17 y [72] which is similar to the maximum age of 20 y that we documented for stoplight parrotfish.

Two previous peer-reviewed studies have reported on population demographics and growth for stoplight parrotfish that included age estimates from otoliths [64,66]. One study examined small-scale demographic variation for stoplight parrotfish from inshore and offshore reefs in the Florida Keys at depths of 2–6 m through comparison of von Bertalanffy growth functions fitted to size-at-age plots for a total of 176 individuals [66]. The maximum age documented in the Florida study was 8 y from an initial color phase individual; the maximum age documented for a terminal color phase individual was 4 y [66]. The second study from the western Atlantic that reported on otolith-based ages for stoplight parrotfish obtained samples from four localities that extended across 14° latitude (San Blas, Panama; Los Roques, Venezuela; Barbados; Lee Stocking Island, Bahamas) [64]. That study documented a maximum age of 9 y and noted that initial color phase individuals achieved similar maximum ages as terminal phase fish [64]. The sample sizes of stoplight parrotfish aged across the four localities ranged from 82–118 individuals. The second study indicated that “the majority of *Sparisoma viride* sampled for age analysis were speared in shallow areas of reef habitats, although an effort was made to collect terminal-phase individuals across the entire depth range occupied” [64]. From this description it was not apparent what depths were actually sampled for fish included in the age analysis (although later in the methods it was indicated that abundance estimates were made at sites ranging in depth from 2–15 m) [64].

The previous two studies differ from the current U.S. Caribbean collaborative research program on parrotfish population demographics (that supplied the samples for age validation in the current study) in a few major ways that may aid in explaining differences in the maximum documented ages among studies. First, the U.S. Caribbean research program has resulted in estimated ages for over 1700 archived stoplight parrotfish samples collected by the USCA FishCon Lab in collaboration with U.S. Caribbean fishers with a variety of gear types (cast nets, bag seine, spear, trammel-net, traps). This large total number of samples and the diversity of gears used, relative to the previous two studies, may have increased the likelihood that older individuals were collected from the U.S. Caribbean. Second, the depth range for the fisheries-dependent samples collected from the U.S. Caribbean by the USCA FishCon Lab was extremely broad. In the U.S. Caribbean, STX spear fishers typically target “plate-sized” initial and terminal color phase stoplight parrotfish at depths ranging from 4–23 m (G. Martinez, STX commercial fisher, personal communication) and STT/J trap fishers commonly catch individuals of both color phases at depths of 25–40 m (J. Magras, STT/J Fisherman’s Association, personal communication). Observations by fishers on depths at which stoplight parrotfish were obtained for use in the current study are corroborated by scientific diver surveys conducted in STX and STT/J [73,74]. For example, fish visual census results from the Marine Conservation District (MCD) of STT/J waters at sites ranging in depth from 28–42 m (average depth of census sites = 36.5 m) ranked stoplight parrotfish as the second most common fisheries species observed (queen parrotfish ranked first) [74]. The broader range of depths from which stoplight parrotfish of both color phases were captured in U.S. Caribbean waters may have increased the likelihood that we obtained older individuals compared to the other two studies.

The oldest queen parrotfish analyzed in the current study for Δ^14^C was a terminal color phase male from STX with an age of 16 y. Interestingly, the oldest queen parrotfish we have aged using the validated ageing method from this study was a 21 y old terminal color phase male (410 mm FL) from Bermuda [69]. A maximum age of 21 y for queen parrotfish is similar to the longevity of 20 y noted for this species by Comeros-Raynal et al. [75]. The focus of Comeros-Raynal et al. [75] was to evaluate the likelihood of extinction for 179 known species of parrotfish and surgeonfish through analysis of species-specific data and is the only other peer-reviewed study that provided an age estimate for queen parrotfish. The longevity estimate of 20 y was reported in a supplemental table, but the original source of the estimate was not provided [75]. A few recent studies from the western Atlantic have reported on maximum ages for other *Scarus* species that also exceed 20 y; greenback parrotfish *Sc*. *trispinosus* has a maximum estimated age of 22 y [76] and midnight parrotfish *Sc coelestinus* has a maximum estimated age of 31 y [20].

Sexual identities associated with parrotfish color phases include: female initial phase, primary male initial phase, secondary male initial phase, primary male terminal phase, secondary male terminal phase, and sexually transitioning initial color phase. Robertson and Warner [13] speculated that primary males and large initial phase males may channel more energy into growth in early life; differences in growth rates may exist among the various sexual identities within a population, but ultimately this can only be verified with size-at-age information combined with gonad histological analysis [20]. For many parrotfish species in the Caribbean, male sexual identities are associated with particular mating behaviors and reproductive strategies. For example, males in the terminal color phase from stoplight, redband, yellowtail, princess, striped, and queen parrotfish species are mostly territorial, form harems, and utilize pair spawning [13,17,77–79]. Initial color phase males do not appear to hold territories, but rather employ several spawning behaviors and mating strategies that relate to interfering with pair spawning of territorial males and also display group spawning [13,80–82]. These divergent male strategies may correlate with differences in growth rates due to variations in energetic investment towards reproductive output versus somatic growth. With validated ageing methods combined with histological analysis of gonads, we can further evaluate these complex parrotfish sexual ontogenies and document sex-specific growth patterns. Moreover, the age distribution of reproductive effort in parrotfishes is not well understood, but could be examined more fully, which will ultimately aid in evaluating population health of individual species and in employing management strategies geared towards sustainable fisheries practices in the U.S. Caribbean and elsewhere. Analyses of population age structure, growth, and reproductive biology for each of the seven U.S. Caribbean parrotfish species sampled as part of our on-going collaborative life history research program is underway and will ultimately provide a more detailed understanding of species-specific differences in growth related to sexual identities and reproductive strategies.

### Western Atlantic *Scarus* and *Sparisoma* species: updated longevities

Fish longevity (life span/maximum age attained) is a key parameter for estimation of natural mortality and survivorship [83] and in computing estimates related to potential lifetime reproductive output [84]. Several studies that investigated demographic patterns of parrotfishes from the Pacific and Atlantic noted that most parrotfishes are relatively short-lived [15,22,64–66]. Molina-Ureña [63] conducted an extensive literature review on biology and ecology of Atlantic parrotfishes and noted that lifespan of Atlantic species ranged from 3–9 y, although since then, additional studies from the Caribbean have documented much older longevities for many species (Table 3). Therefore, it is important to re-evaluate our understanding concerning overall patterns of longevity for parrotfishes from the western Atlantic.

**Table 3:** Summary information for *Scarus* and *Sparisoma* parrotfish species in the western Atlantic.

Species	Region	Size group	Fisheries status	General L_max_ (mm)	Caribbean L_max_ (mm)	t_max_ (y)
***Scarus* species**						
Midnight *Sc coelestinus*	West Atlantic	Large	Protected	770 TL^1, 2^	656 FL/728 TL Bermuda^3^	31^3^
Blue *Sc coeruteus*	West Atlantic	Large	Protected	900 TL^1^/1200 TL^2^		16^1^
Rainbow *Sc guacamaia*	West Atlantic	Large	Partially protected; Incidental	1200 TL^1,2^	650 FL/692 TL^4^	16^1^
Striped *Sc iseri*	West Atlantic	Small	Incidental	350 TL^1^/270 TL^2^	247 TL^12^	8^1^, 10^12^
Princess *Sc taenopterus*	West Atlantic	Small	Commercial	350 TL^1^/300 TL^2^	314 TL^3^/377 FL^5^	11^3^
Greenback *Sc trispinosus*	Southwest Atlantic	Large	Commercial^6,7^ Endangered^6^	700 TL^1^/860 FL^7^	-	22^7^
Queen *Sc vetula*	West Atlantic	Medium	Commercial	500 FL^1^/500 TL^2^	420 FL^5^ 456 FL/521 TL Bermuda^4^	21^4^
Zelinda’s *Sc zelindae*	Southwest Atlantic	Medium	Commercial^6^	332 SL^1^	-	12^1^
***Sparisoma* species**						
Reef *Sp amplum*	Southwest Atlantic	Large	Commercial^6^	700 TL^9^	-	17^9^
Greenblotch *Sp atomarium*	West Atlantic	Small	Incidental	250 TL^1^/150 TL^2^	-	3^1^
Redband *Sp aurofrenatum*	West Atlantic	Small	Commercial	280 TL^1, 2^	257 FL/296 TL^12^	7^1^, 11^12^
Redeye/Gray *Sp axillare*	Southwest Atlantic	Medium	Commercial^6^	430 TL^2^/420 TL^11^	-	12^11^
Redtail *Sp chrysopterum*	West Atlantic	Medium	Commercial	460 TL^1. 2^	394 FL^12^/500 FL^5^	5^1^, 13^12^
Agassiz’s *Sp frondosum*	Southwest Atlantic	Medium	Commercial^6^	345 SL^1^/370 TL^10^	-	12^1^; 9^10^
*Sp griseorubrum*	Central west Atlantic	Small	Unknown	270 TL^1^	-	
Bucktooth *Sp radians*	West Atlantic	Small	Not fished	200 TL	-	
Trinidade *Sp rocha*	Southwest Atlantic	Medium	Commercial^6^	305 SL^8^	-	
Yellowtail *Sp rubripinne*	West Atlantic	Medium	Commercial	478 TL^1^/480 TL^2^	345 FL^4^/395 FL^5^	7^1^, 14^4^
*Sp tuiupiranga*	Southwest Atlantic	Small	Unknown	154 SL^1^	-	
Stoplight *Sp viride*	West Atlantic	Medium	Commercial	500 TL^1^/640 TL^2^	433 FL^4^/505 FL^5^	20^4^; 20+^13^

^1^ Comeros-Raynal et al. [75]; ^2^ Robertson and Van Tassell [85]; ^3^ Jones et al. [20]; ^4^ Current study efforts; ^5^ Stevens et al. [86]; ^6^ Cunna et al. [87], Roos et al. [8], Roos et al. [88]; ^7^ Freitas et al. [76]; ^8^ Pinheiro et al.[89]; ^9^ Xavier [72]; ^10^ Lessa et al. [90]; ^11^ Gaspar [91]; ^12^Rivera Hernández and Shervette [69]; ^13^Van Rooij and Videler [70].

A total of 20 parrotfish species from the genera *Scarus* and *Sparisoma* occur in the Western Atlantic (Table 3); 12 species mainly occur throughout waters of the Caribbean, GOM, and Bermuda (West Atlantic); seven are endemic to waters of Brazil (Southwest Atlantic); and one species is mainly isolated to waters of Venezuela (Central west Atlantic). At least 15 of these species are targeted or caught incidentally in reef fisheries landings (Table 3). These 20 species can be roughly categorized into three groups based on maximum lengths attained. Midnight parrotfish *Scarus coelestinus*, blue parrotfish *Sc coeruleus*, rainbow parrotfish *Sc guacamaia*, greenback parrotfish *Sc trispinosus*, and reef parrotfish *Sparisoma amplum* attain maximum lengths of 700–1200 mm TL and comprise the “Large” parrotfish size group of the western Atlantic (Table 3). The “Medium” size group includes queen parrotfish, Zelinda’s parrotfish *Sc zelindae*, redeye parrotfish *Sp axillare*, redtail parrotfish, Agassiz’s parrotfish *Sp frondosum*, Trinidade parrotfish *Sp rocha*, yellowtail parrotfish, and stoplight parrotfish which attain maximum lengths ranging from 410–610 mm TL (Table 3). The “Small” size group contains striped parrotfish, princess parrotfish, greenblotch parrotfish *Sp atomarium*, redband parrotfish, the endemic Venezuelan parrotfish *Sp griseorubrum*, bucktooth parrotfish *Sp radians*, and *Sp tuiupiranga* with maximum sizes ranging from 180–380 mm TL (Table 3).

Results from the current study indicated that opaque zone counts from sectioned sagittal otoliths of *Sparisoma* and *Scarus* species provide accurate age estimates for Caribbean parrotfishes. Detailed documentation of age, growth, and reproductive biology for each of the seven species landed in the U.S. Caribbean reef fisheries has so far resulted in obtaining otoliths and gonads for over 5,800 samples (over 90% of the samples were fisheries-dependent collections) from 2015–2022 as part of the on-going collaborative efforts to fill in critical life history information gaps for these species [69]. Maximum ages for these and other Caribbean parrotfishes from the recent literature provided updated estimates for longevity of western Atlantic *Scarus* and *Sparisoma* species (Table 3) indicating that many parrotfishes in this region are relatively long-lived. Parrotfishes in the “Large” group with previously unknown or updated longevity estimates include (Table 3): midnight parrotfish with a maximum age of 31 y [67], greenback parrotfish with a maximum reported age of 22 y [76], and reef parrotfish with a maximum age of 17 y [72]. Species in the “Medium” group with updated longevities include (Table 3): queen parrotfish attaining an estimated maximum age of 21 y [69], redeye parrotfish with a maximum age of 12 y [91], redtail parrotfish with a maximum age of 13 y [69], yellowtail parrotfish attained a maximum age of 14 y [69], and stoplight parrotfish with a maximum age of 20 y [69]. Maximum reported ages for parrotfish in the “Small” group have also been recently reported or extended: princess parrotfish can live up to at least 11 y (Fig 2B) [67], striped parrotfish up to 10 y [69], and redband parrotfish longevity was extended to 11 y [69] (Table 3).

## Conclusions

Parrotfishes are integral to the ecosystem function and maintenance of shallow water coral reefs in addition to contributing to reef fish fisheries for locals in the Caribbean. Fisheries management jurisdictions of the Caribbean often lack resources to enforce wide-ranging compliance with regulations. For management efforts to succeed in the region, a majority of local fishers must be supportive and willing to comply with regulations which is more likely when fishers are included in a stock assessment process that utilizes robust scientific evidence collected in collaboration with fishers, to evaluate the health of fish stocks [92,93]. Scientifically rigorous stock assessments require regional species-specific information on age-based key life history parameters, derived from validated age estimation methods, including population age structure, growth rates, maturity, sexual transition, longevity, and reproductive output [6,9–12]. The Δ^14^C validation of age estimation methods for Caribbean parrotfishes from the current study means that these parameters can now be obtained for use in the sustainable management of Caribbean parrotfish species.
